# Barriers to health care utilization among patients with type 2 diabetes living in slums: a qualitative study from providers' perspective

**DOI:** 10.1186/s41256-023-00296-0

**Published:** 2023-04-21

**Authors:** Fawzieh Ghammari, Rahim Khodayari-zarnaq, Habib Jalilian, Masumeh Gholizadeh

**Affiliations:** 1 Department of Health Policy and Management, School of Management and Medical Informatics, Tabriz University of Medical Sciences, Tabriz, Iran; 2 Department of Health Services Management, School of Health, Ahvaz Jundishapur University of Medical Sciences, Ahvaz, Iran

**Keywords:** Health care utilization, Slums, Type 2 diabetes, Qualitative study

## Abstract

**Background:**

Due to slum dwellers' deprivation, they are more likely to develop Type 2 Diabetes (T2D) and its complications. Type 2 Diabetes is a long-life disease that requires continuous health care utilization. One of the negative outcomes of slum-dwelling is health care underutilization. Therefore, this study aimed to understand barriers to health care utilization among those with T2D living in Tabriz slums, Iran, from the perspective of healthcare providers, in 2022.

**Methods:**

A phenomenological approach was used in this study. Purposive sampling for conducting in-depth interviews was used to select 23 providers consisting of general practitioners, midwives, nutritionists, and public health experts. We conducted a content analysis using the 7 stages recommended by Colaizzi. We used four criteria recommended by Lincoln and Guba for ensuring the research’s trustworthiness.

**Results:**

Three main themes and 8 categories were developed. Three main themes are 1) health care provision system barriers, including four categories: lack of motivation, non-availability of facilities and doctors, poor relationship between patients and providers, and disruption in the process 2) coverage problems, including two categories: insurance inefficiency, and limited access, and 3) contextual barriers, including two categories: environmental problems, and socioeconomic barriers.

**Conclusions:**

Recommendations are presented in three levels to improve implementation. The health care system needs to modify the payment methods, Patients-providers relationship improvement, and increase the number of providers. Insurance organizations should consider sufficient coverage of costs for slum-dwellers with T2D and expand the benefits package for them. Government should consider infrastructure upgrading in slums to eliminate barriers related to slum-dwelling. Overall, health care utilization promotion needs intersection cooperation.

## Background

Globally, over a billion people lived in slums in 2016, and this number is expected to increase [1]. According to the United Nations, slums are characterized by poor housing conditions, overcrowding, and a lack of basic health and welfare facilities. Due to these conditions, adverse health outcomes occur, including underutilization of healthcare [2]. It also disrupts lifestyle behaviors for diseases such as type 2 diabetes (T2D) [3]. Managing and controlling T2D require continuous health care utilization throughout lifetime [4]. Approximately 347 million people are suffering from T2D around the world. The number of deaths caused by this disease is expected to double by 2030 [5]. More than 75 percent of diabetes patients live in developing countries, and the trend is on the rise [6]. In developing countries, lifestyle changes have contributed to the rise of obesity as one of the most important causes of type 2 diabetes. [7]. There are several complications associated with T2D. As a result, developing countries face financial difficulties [8].

People living in slums are more likely to develop T2D and its complications [9, 10]. In slum-dwellers, specific behaviors and circumstances increase the risk of T2D. Results of a study among slum-dwellers in Kenya found the weighted prevalence of behavioral factors increasing non-communicable diseases (NCDs), including unhealthy diet (57.2%), poor physical activity (14.4%), alcohol abuse (10.1%), and tobacco use (12.4%) [11]. A study in Pune slum, India showed that the number of those at risk for T2D is high due to poor education, low exercise, high waist circumference, and low socioeconomic status [12]. Results of a study in Brazil showed the prevalence of T2D is nearly two-fold among slum-dwellers compared to the general population (10/1% vs 5/2%) [13]. In contrast, slum-dwellers are less likely to have health care utilization. A study in Vietnam shows that, compared to the rest of the population, slum-dwellers use fewer health services for NCDs such as diabetes (21/4% vs 26/9%) [14]. Considering the lifelong nature of diabetes, as well as more need for health care utilization among slum-dwellers, health care utilization in patients with T2D plays a critical role in preventing diabetes deterioration, its complications, and economic hardship arising from it.

Recently, the number of slum-dwellers in Tabriz, one of Iran's metropolises, has increased. According to municipal officials, more than 400,000 people live in Tabriz's slums. More than 13,000 people with T2D live in Tabriz slums. Despite efforts of Iran's health ministry for universal health coverage (UHC) expansion, as well as launching Iran's Package of Essential Non-communicable (IraPEN) to increase access to care and prevention of NCDs including T2D, Tabriz slum dwellers' health services underutilization is more frequent than Tabriz residents’ because of high costs (7/2% vs 3/3%) [15, 16]. In this study, Health care providers were selected to understand why underutilization because they are directly involved in health care provision, and know health system problems well, as well as slum-dwellers problems for T2D self-management. Identifying these challenges can help improve planning and implementation in slums and decrease the health and economic complications of T2D. Therefore, we conducted a qualitative study to understand barriers to health care utilization for patients with T2D from the perspective of healthcare providers in Tabriz's slums.

## Methods

### Study design

An interpretative paradigm with a phenomenological hermeneutic approach informs our research [17]. Phenomenology is a subjective and systematic approach to describing the deepest experiences in life [18]. This approach does not control contextual variables, allowing researchers to understand the phenomenon more deeply [19, 20]. This phenomenological study was conducted using content analysis on in-depth interviews. In Content analysis, the meaning of data is organized and extracted to achieve realistic inference [21]. In terms of researcher characteristics and reflexivity, out of 4 researchers, 3 had research experience about health-seeking behavior and forgone care among those with T2D in Tabriz. These studies weren’t directly related to slum-dwellers. The interviewer had no research experience about T2D and health centers located in slums.

### Participants, sampling, and data collection

Purposive sampling was used to select participants. Therefore, we selected information-rich providers through some inclusion criteria [22]. Inclusion criteria were having career experience of over 5 years in slums for ensuring a deeper understanding of slums' problems. These providers were responsible for providing health care to diabetic patients and the rest of the population. They were general practitioners, midwives, nutritionists, or public health experts.

There are 13 health centers in Tabriz slums. We referred to health centers located in slums and obtained information about providers through the head of centers. 1–2 providers were selected for each health center. On each day two interviews were conducted.

For a deeper understanding of the asked phenomenon, Polkinghorne suggests interviewing 5 to 25 people with full experience [23]. In total, 23 of 130 providers who worked in health centers located in slums were interviewed. The duration of each interview was between 40 and 60 min. The interviews were conducted in the Persian language. Data were collected and analyzed from 5 April 2022 to 30 September 2022.

Data collection was conducted via face-to-face in-depth interviews by a researcher. All interviews were recorded using a tape recorder after the written consent form was obtained and the study process explained. At the same time, facial expressions were considered and recorded. Facial expressions were considered semantic units. On the same day, data were transcript word for word by a researcher.

To cover the purpose of the study, four researchers discussed guiding questions. Finally, the guiding question was: “according to your experience in the slums, what are barriers to health care utilization among people with T2D living in slums?”.

The next questions were to clarify the providers' sentences and more discussion.

### Data analysis

We used 7 stages recommended by Colaizzi to analyze phenomenological methods [24]. These stages are 1) reading the transcript many times for data familiarity 2) extracting sentences that are directly related to the phenomenon 3) categorizing and creating themes based on semantic similarity 4) repeating stages 1–3 for each interview and creating themes based on semantic formulated 5) comprehensive collecting for stages 1–4 6) Summarizing of comprehensively collected data to identify the basic structure of the phenomenon 7) Discussion with experts to ensure the credibility of the data.

The data analysis was performed by two researchers, separately and manually. The transcripts were read many times to obtain a general sense of the text. The significant statements related to the phenomenon were extracted. Formulated meanings were extracted from the significant statements. In the next stages, codes, categories, and themes were organized. Two other researchers reviewed them. Disagreements were resolved by discussion between four researchers. Four researchers recognized the saturation of data after the 21st interview. After that, two additional interviews were conducted to increase credibility. The final codes, categories, and themes were developed during 4 h of discussion among four researchers.

### Trustworthiness

Four criteria (credibility, transferability, dependability, and confirmability) recommended by Lincoln and Guba were used to ensure the data’s trustworthiness [25]. We checked the results with participants to ensure credibility. We have attempted to provide the details of the study and to make the analysis process transparent to enhance transferability. To ensure dependability, the results were discussed with two researchers outside the study. To improve confirmability researchers explained their reflexivity. This study has followed the guidelines recommended by the standards for reporting qualitative research (SRQR) checklist [26].

## Results

Among 23 providers, there were 13 midwives and 4 public health experts. Two of the providers were nutritionists, and 4 were general practitioners. All the providers were women. Providers’ career experience was between 5 and 8 years. The number of initial codes was 350. After merging the initial codes, the number of them decreased to 20 final codes. Eight categories were developed from the final codes. Finally, three main themes were developed. Three identified main themes are 1) Health care provision system barriers, 2) Coverage problems, and 3) contextual barriers.

### Theme 1: Health care provision system barriers

The health system has a prominent role in patient health care utilization. The health system impact utilization from different dimensions. This theme is including 4 categories and 12 codes. Four categories include lack of motivation, non-availability of facilities and doctors, poor relationships between patients and providers, and disruption in the process.

### Category 1.1: lack of motivation

Providers are being plagued by apathy. They stated that their livelihood primary needs are unmet. Therefore, they aren’t willing to health care provision well. They are constantly comparing themselves with the other workers who despite the same educational rank receive higher wages. There is no justice in payments between public and public–private partnership (PPP) workers. They repeatedly said: “Why I should do my best?” (Participant 15).

The majority of providers have been hired under a volume work contract through PPP. They have to take care of a certain number of people. Payments are based on the number of people who received care. Salaries are paid late and incompletely. Providers stated that they are not willing or able to do their duties completely. “With this salary, we can't make a living. Making both ends meet is challenging for us. We conduct work equivalently with wages” (Participant 1).

According to providers, their job situation is unstable. There is a constant stress to fire providers. They had to do whatever was to be told without expecting pay change. The number of unemployed experts is high in Iran. Therefore private sector is not under pressure for new provider recruitment and can replace providers. “We have been hired as if we were a slave (anger and laugh). We have no right to object to working conditions. We are to be told to go out” (Participant 6).

The lack of gratitude prevents providers to the provision of high-quality care. “They expect us to do our best (wonder). If you do well, no one will thank you. We have been neglected” (Participant 9).

The providers aren't satisfied with the evaluation system. There is no comprehensive evaluation system that considers concurrently qualitative and quantitative performance. The system just evaluates based on quantitative performance. Providers implicitly stated that they could influence the quality of care by concentrating on reporting. “You have a list of patients. You must fill out the care form. It doesn't matter how they have been cared for. Just and just the number is important (anger)” (Participant 8).

### Category 1.2: Non-availability of facilities and doctors

Health centers in slums suffer from a lack of equipment. Equipment such as a glucometer and barometer are needed for regular control of T2D. It isn't possible to take care of patients without proper equipment. “We call the patients. We have to follow up with patients. We have to follow them once every three months. They come, once, twice, three times, but this barometer (AH!) doesn't work and glucometer doesn't work” (Participant 11).

According to the providers, patients want to go to health centers, but there are not enough doctors. Sometimes patients go to health centers to see a general practitioner several times. Doctors are not willing to work in slums. Salaries are low and doctors can go to other centers. “Patients are coming. The doctor isn’t here. This center covers 70,000 people but has only 1 doctor. We can weigh patients and give them advice about weight control, advise them about medicine regularly, regular testing, and so on. Repetitive advice. In fact, without doctors, they don’t receive any services. They curse us (sad). They go and they don’t come back” (Participant 6).

### Category 1. 3: Poor relationships between patients and providers

There is no appropriate relationship between patients and providers. “We cannot even look at patients' faces. The health system has made us so” (Participant 7).

Health centers pay attention to outputs, namely the number of patients cared for. The proper utilization will reduce without a good relationship between patient and provider. Until the health system will not clear on the importance of communication and will not educate providers, the appropriate utilization will remain an unachieved goal. One of the participants says: “A good relationship isn’t our duty” (Participant 18).

Poor relationships make it impossible to understand patients' needs and demands. Patients are discouraged when they can’t receive the needed health care. Educations are unilateral and general and without attention to patients’ needs. “We take care without attention to actual needs and demands… Everything is unilateral” (Participant 15). Therefore, Patients don't desire to continue care in health centers.

### Category 1.4: Disruption in the process

There is no good collaboration between providers. There is a conflict between doctors and non-doctors providers. A midwife says: “Doctors refer patients to us… They want just to get rid of patients” (Participant 15).

Experts believe that the actions of providers are fragmented and there is no integrity between them. Actions are not aligned together. “Everyone carries out the duty of herself/himself” (Participant 20).

Work overload affects the quality of care and consequently appropriate health care utilization. The health system imposes much duty on providers. They have to regularly provide care for different ages and gender groups. A public health expert says: “Not only patients with type 2 diabetes but also pregnant women, children, young people, and the elderly should have been cared for… We can't provide adequate care” (Participant 13).

The integrated health system known as SIB is a key barrier to effective health care. The SIB is a system for recording people's health information. Simultaneous care delivery and filling out the care form at the SIB is the main constraint to delivering quality care. In addition, it is time-consuming to complete the care form at SIB. “We have to focus on the SIB. It has to be completed. At the same time, we have to provide service to patients” (Participant 5).

The ministry of health has defined the referral system, but it has not been implemented. Neither patients nor physicians follow the referral system. “Patients go wherever they want, which confuses us. We call them, they say we have received health services, but how? Doctors have no interest in accepting patients. They guide them indirectly to private centers. They don’t provide services for patients (Wonder)” (Participant 3).

### Theme 2: coverage problems

Some barriers to health care utilization are derived from insurer organizations' inefficiency due to insufficient cost coverage and health care provision limits. This theme is including two categories and four codes. Two categories include insurance inefficiency and limited access.

### Category 2.1: insurance inefficiency

Patients have to pay high money when needing necessary services. Some patients overlook receiving care services when aren’t able to pay the costs or when they think services haven’t worth spending money on.

The majority of slum-dwellers are covered by two types of insurance, and some of them aren’t covered by insurance. Social Security Insurance and Iran health insurance are these two insurer organizations. The social security insurance is a public non-governmental organization that covers employees in the formal private sectors, as well as self-employed and voluntary contributors. The social security insurance is the primary insurance in Iran. Social security organizations' hospitals and health centers have no copayment. The number of people covered by social security insurance is more than two-fold compared to Iran health insurance organization (43.475.548 people in 2019). Iran health insurance organization covers unemployed and vulnerable people including slum-dwellers [27]. Therefore, Insured patients by Social Security Insurance are in a better situation in terms of access to health care. This organization has a few hospitals and clinics which provide health care services, freely. But Iran health insurance has neither such centers nor sufficient coverage of costs. “Some patients are without insurance. Patients with Social Security Insurance go to free centers. Insured patients with Iran health insurance come here. But appropriate services aren’t delivered” (Participant 18).

### Category 2.2: limited access

There is a difference between providing services among insurance organizations. Some provide diverse services including free doctor visits and free tests but some cover very limited services. Visitors to free social security insurance treatment centers face long queues. However, lots of them have to wait. As a result, appropriate health care utilization is negatively affected. “Some patients have to wait for 6 months. The disease deteriorates” (Participant 10).

### Theme 3: contextual barriers

Some problems in health care utilization are arising from slum-dwelling. Slum-dwelling contexts have differences from the rest of the city which influence utilization. This theme includes two categories and four codes. Two categories consist of environmental problems and socioeconomic barriers.

### Category 3.1: environmental problems

Sometimes women can’t refer to health care centers due to safety reasons. There is a disturbance for them. Sometimes they have to refer together or with their husbands. “It is the elderly who suffer from the adverse effects of slum living. Bad architecture” (Participant 11).

### Category 3.2: socioeconomic barriers

The slum-dwellers are classified as socioeconomically disadvantaged groups. Poor literacy is one of their characteristics. The patients do not have enough information about their disease. Additionally, they are unaware of the outcomes of underutilization on T2D. “They don't know what will happen if they don't control their disease” (Participant 5).

Most patients residing in slums are poor. They aren’t able to receive expensive services. Free or cheap health services aren’t provided in health centers located in slums. “Visiting a doctor is free. But patients have to provide medicine in the market…They have to conduct the tests in the market. They are expensive in the market. They are slum-dwellers… Some patients will be deprived of appropriate health care” (Participant 9).

**Fig. 1:**
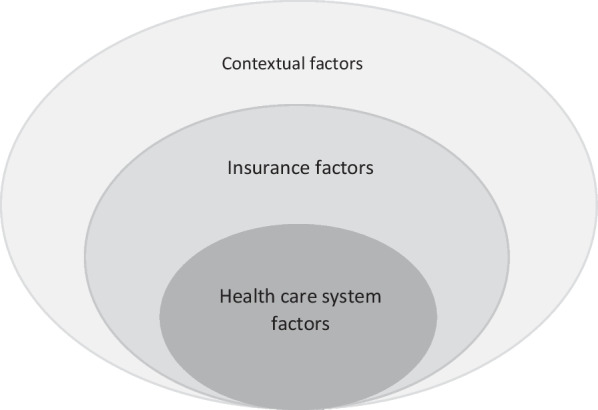
Barriers to health care provision and utilization among those with T2D living in slums

Table 1 illustrates generated three themes. Based on them, we found three influential factors on health care provision and utilization, which are shown in Fig. 1.

**Table 1:** Themes, categories, codes, and formulated meanings

Theme	Categories	Final codes	Formulated meanings
Health care provision system barriers	Lack of motivation	Low wages	It is challenging to manage livelihoods
		Job instability	There is a lack of stability in the employment situation of providers
		Ungratefulness	Health care providers have been ignored
		Lack of comprehensiveness in the Evaluation system	The payment system is based on quantitative performance
	Non-availability of facilities and doctors	Lack of equipment	Handling patients adequately is impossible due to a lack of equipment
		Doctors’ shortage	The number of patients exceeds the number of doctors available to treat them
	Poor relationship between patients and providers	Communication is unimportant	There has been a lack of understanding in the health system about the importance of patient-provider relationships
		Poor understanding of patients’ needs	Communications are more unilateral
	Disruption in the process	Poor collaboration	There is poor cooperation among health system workers
		Work overload	The providers are responsible for a variety of tasks
		Paper work	Filling out forms is the main focus of health care providers
		Defective referral system	Neither patients nor providers are aligned with the referral system
Coverage problems	Insurance inefficiency	High out of pocket	There are high payments by patients
		Insufficient coverage of health costs by the insurance	There is a high out-of-pocket expense
	Limited access	Benefit package limits	Various and required services are not usually provided
		Overcrowding of free treatment centers	Queues are too long, reducing access at the right time
Contextual barriers	Environmental problems	Unsafe environment	Refer to health centers is coupled with disturbs for women
		Unsuitable environment	The slums' architecture is unsuitable for the elderly to refer to health centers
	Socioeconomic barriers	Poor health literacy	There is a lack of information about the disease and its complications among patients
		Poverty	It is difficult to access appropriate health care because of financial barriers

## Discussion

We extracted three factors that are the barriers to health care provision and utilization including health care system factors, insurance, and contextual factors.

Health System-related factors can affect providers’ performance. One of these factors is motivation. There are several aspects to motivation. One of them is wages of health care providers. Provider payment methods can change the behavior of the provider. Changing provider behavior can happen in three ways: resource changing, service changing, and cost changing [28]. Another aspect is the importance of gratitude in the service delivery process. Gratitude affects providers' behavioral, psychological, and attitudinal aspects [29]. A study in the United Kingdom found that quality-of-care measures declined immediately after financial incentives were removed [30]. Therefore, the government and health system should consider fair payment system and resolve the wage gap between public and private providers.

The health system does not pay attention to providing essential equipment and providing sufficient doctors for diabetic patients. Non-availability of health services affects health-seeking behavior and decreases efficient diabetes management [31]. Innovative ways for financing the provision of equipment and doctors are necessary. The use of domestic donors, as well as international aid, can be beneficial.

The importance of a good relationship has been repeatedly stated by providers. Previous studies have shown that good communication affects diabetes management [32, 33]. A good relationship is a missing link to improving health care utilization. According to providers an appropriate relationship between providers and patients can lead to satisfaction and trust. As a result, the health system should improve communication between patients and providers.

Provider collaboration is essential for improving care and preventing fragmentation and errors [34]. There is a lack of collaboration between health care providers in providing patient services. The health experts confirmed that doctors have more decision-making power than health experts. They stated that doctors don't work and just refer patients to specialists. Integrated service delivery and system thinking should be considered in health centers.

There is a negative impact on the quality of care when there is work overload [35]. Providers deal with a large population. Recently, providers should be providing care to a wide range of ages. Diabetes patients aren't getting enough attention from providers due to a lack of time. As a result, the health system should increase the number of providers.

Patients' information is maintained, recorded, and updated in the integrated health system (SIB). According to participants, the SIB damages the proper relationship and reduces the quality of care. Providing care and filling out the care form are conducted simultaneously. SIB's care form adds to providers' workload. Due to work overload, technology indirectly contributes to psychological detachment from work [36]. Therefore, the health system must use health information technology in a way that doesn't damage the appropriate delivery of services. There can be a clearer division of work between health care providers.

Referral systems are defective, making the health care market confusing. The referral system is influenced by technology, processes, organizational, and patient-related factors [37]. Therefore, it is necessary to pay special attention to factors affecting the referral system to improve its performance.

Some slum dwellers are not insured. The results of a study show uninsured patients with T2D are less likely to appropriate health care utilization [38]. The Iranian insurance market consists of four main organizations. These insurer organizations include Social Security Insurance, Iran Health Insurance, Armed Forces Health Insurance, and Imam Khomeini Relief Committee Health Insurance. Covered populations in each organization have different benefits packages. According to providers, the majority of slum-dwellers are covered by Social Security Insurance and Iran Health Insurance. Iran Health Insurance organization tries to cover the uninsured. Results of a study in Iran indicated health care utilization and diabetes management are influenced by the type of insurance [39]. The coverage of health expenses by Iran Health Insurance is very low. Insured people can benefit from free health care through Social Security Insurance, but long queues prevent them from utilizing these services at the proper time. There is a need for equal package benefits between different insurer organizations. It is also necessary to increase the number of free health centers with minimum waiting time.

Contextual factors have an essential role in the underutilization among slum-dwellers. The importance of a safe and secure environment on regular referrals to health centers and consequently health care utilization has been shown in previous studies [40, 41]. In addition, Slum apartment buildings and street textures are messy, and referring to health centers is difficult. One of the key recommendations for improving health care utilization is to upgrade slum infrastructure.

We should not overlook the importance of literacy. In particular, health literacy may influence health care utilization[42]. Also, health literacy leads to appropriate diabetes management [43]. Considering low education among slum-dwellers, peer groups are recommended for improving diabetes management [3, 31].

Poverty is a barrier to diabetes management among patients from lower socioeconomic positions [39, 44]. Some slum dwellers cannot afford medical tests and medicine. Governmental health centers are their only source of healthcare. While these health centers face several challenges in delivering services. To improve health care utilization among those with type 2 diabetes living in slums, the government and health system must consider payment exemptions for necessary services.

This study has some limitations. The participants were relatively conservative. Due to the purposive sampling technique and insufficient sample size, the findings of this study may not be generalized to other slums.

## Conclusions

There are three levels of recommendations for improving implementation. The first and most important level is related to the health care provision system. Health centers in slums need innovative financing approaches to provide manpower, equipment, and other resources. The health system should consider reviewing the wages and evaluation systems. The service delivery process such as documentation needed to be modified. At the second level, insurer organizations, especially Iran Health Insurance, must consider sufficient coverage of costs for those with T2D living in slums and expand the benefits package for them. At the third level, namely contextual factors, upgrading of infrastructure should be considered by the government until the slum-dwellers could regularly refer to health centers.

## Data Availability

All data have been applied in the article.
